# An optimized method for counting dopaminergic neurons in zebrafish

**DOI:** 10.1371/journal.pone.0184363

**Published:** 2017-09-07

**Authors:** Hideaki Matsui, Atsushi Sugie

**Affiliations:** 1 Department of Neuroscience of disease, Center for Transdisciplinary Research, Niigata University, Niigata-shi, Niigata, Japan; 2 Brain Research Institute, Niigata University, Niigata-shi, Niigata, Japan; National Center for Toxicological Research, UNITED STATES

## Abstract

In recent years, considerable effort has been devoted to the development of a fish model for Parkinson’s disease (PD) to examine the pathological mechanisms of neurodegeneration. To effectively evaluate PD pathology, the ability to accurately and reliably count dopaminergic neurons is important. However, there is currently no such standardized method. Due to the relatively small number of dopaminergic neurons in fish, stereological estimation would not be suitable. In addition, serial sectioning requires proficiency to not lose any sections, and it permits double counting due to the large size of some of the dopaminergic neurons. In this study, we report an optimized protocol for staining dopaminergic neurons in zebrafish and provide a reliable counting method. Finally, using our optimized protocol, we confirmed that administration of 6-hydroxydopamine (a neurotoxin) or the deletion of the PINK1 gene (one of the causative genes of familiar PD) in zebrafish caused significant reduction in the number of dopaminergic and noradrenergic neurons. In summary, this method will serve as an important tool for the appropriate evaluation and establishment of fish PD models.

## Introduction

Parkinson’s disease (PD) is characterized by movement disorders, degeneration of the dopaminergic neurons, and inclusion bodies called Lewy bodies [1]. Various animal models have been used to investigate the mechanisms of PD [2]; however, the pathophysiology of this disease remains largely unknown, limiting the development of therapeutic treatment strategies. Small fish, including zebrafish and medaka, have been used as animal models in basic science research because of their small body size, large egg production, rapid development, low husbandry costs, and transparency during embryogenesis [3,4]. Current advances in genetic engineering and in disease genetics have increased the utility of these fish to study neurological diseases such as PD [5–8].

We and others have previously identified networks of dopamine neurons in both zebrafish [9] and medaka [10] that possess structures and functions that are conserved among vertebrates [11]. For example, posterior tubercular/hypothalamic dopamine neurons in teleosts project to the spinal cord and require transcriptional factors similarly to the A11 neurons in mice [12–16]. In addition, similar to the mammalian A12 neurons, teleost dopaminergic neurons in the anteroventral part of the preoptic area control the release of luteinizing hormone from the anterior pituitary [17,18]. Dopaminergic neurons in the teleost prethalamic area appear to be homologues of A13 dopaminergic neurons in mammals [19]. Importantly, the cluster of dopaminergic neurons in the teleost posterior tuberculum may contain A9-like neurons found in the substantia nigra pars compacta (SNc) of mammals. Although there are far fewer dopaminergic neurons in the zebrafish compared to mice, (mouse substantia nigra: 8,000–12,000 [20]; zebrafish DC2 and DC4: approximately 25), the teleost dopaminergic neurons in the posterior tuberculum exhibit similar cellular vulnerability as the mammalian A9 neurons [21,22]; may contribute to spontaneous swimming movements [23]; and some project axons to the striatum [16,24]. Furthermore, two transcriptional factors used to identify midbrain dopaminergic neurons in mammals, Pitx3 and Lmx1b, are also expressed in the fish diencephalon [25]. Similarly, Nurr1, required for differentiation of midbrain dopaminergic neurons in mammals, is responsible for the expression of tyrosine hydroxylase (TH) in the teleost dopaminergic neurons within the posterior tuberculum [26]. Finally, noradrenergic neurons are found in the locus coeruleus and the caudal medulla in both teleosts and mammals [27], and the noradrenergic neurons in the locus coeruleus are vulnerable to cell death in PD patients and in various PD models [11,28–30]. In summary, the many similarities between fish and mammalian dopaminergic neurons make fish a potentially useful model for elucidating PD pathogenesis.

To establish and evaluate PD models, it would be important to be able to accurately count the dopaminergic neurons in the posterior tuberculum and/or noradrenergic neurons in the locus coeruleus. There have been two methods reported for counting the dopaminergic neurons in the posterior tuberculum. The first method is to obtain serial paraffin sections, perform immunohistochemistry to label dopaminergic and noradrenergic neurons, and then to count the total number of TH^+^ cells [5,6,8,10]. However, there are several technical difficulties associated with this protocol, restricting its application: 1) the total number of TH^+^ neurons in the posterior tuberculum or the locus coeruleus is relatively small [10], precluding stereological estimation; 2) the use of paraffin or frozen sections results in double counting due to the large size of these neurons [10]; and 3) cutting perfect serial sections without losing any sections requires a high level of technical skill. The second method involves whole mount staining of the dopaminergic neurons [7,31], which works in larval fish but not in adult fish. Considering that PD is an age-dependent disease, the ability to evaluate target neurons in adult fish would be necessary.

Here, we propose a reliable and easy method for counting dopaminergic neurons in the adult zebrafish posterior tuberculum by using thick microsliced sections, enhanced antigen retrieval, and confocal microscopy. This method allows for the proper counting of dopaminergic neurons without the issue of double counting, and would be useful in the development of future PD fish models.

## Materials and methods

### Ethics statement

All the animal experiments were conducted in compliance with the protocol, which was reviewed and approved by the Institutional Animal Care and Use Committee and by the President of Niigata University (Permit Number: #28 Niigata Univ. Res.367-1 and #28 Niigata Univ. Res.483-1).

### Zebrafish maintenance

Zebrafish (AB) were raised and maintained under a 14-h light/10-h dark cycle at 28°C according to standard protocols [32,33]. From 5 days post-fertilization, fish were fed brine shrimp at 9:00 a.m. and powdered feed (Kyorin, Himeji, Japan) at 12:00 p.m. Only male fish were used for this study.

### Microinjections

Glass capillaries (GD-1; Narishige, Tokyo, Japan) were pulled into microinjection needles by using a vertical needle puller (PC-10; Narishige). These needles were used in an IM-31 microinjector (Narishige) equipped with a YOU-1 micromanipulator (Narishige). To generate PINK1-deficient zebrafish, guide RNA (target sequence: CCGGCCGGTACCGCTTCTTCAGG, 25 ng/μl) and Cas9 protein (0.6 μg/μl; New England Biolabs, Ipswich, MA) were mixed with phenol red (2%) and co-injected into one-cell stage fish embryos according to previous reports [34,35]. The F1 generation and subsequent generations were genotyped using PCR (forward primer: GGTCCGTAAAAGCCTTCAGA, reverse primer: CTGGTTCTGTCCTCCTCCTG) and direct sequencing (sequencing primer: CTGGTTCTGTCCTCCTCCTG). Heterozygous mutant fish were crossed to obtain homozygous mutant (PINK1-KO) and control fish. For both genotypes, five fish per group were subjected to immunofluorescence studies at 4 months.

### 6-hydroxydopamine (6-OHDA) treatment

Cerebrospinal fluid (CSF) injections of 6-OHDA were performed as described previously [36]. Briefly, the micropipette for CSF injection was made from a 1-mm diameter glass capillary tube (GD-1; Narishige) using a needle puller (PC-10; Narishige). The tip of the micropipette was broken by gently pressing it against the bottom of a 1.5-ml tube. Twelve-month-old zebrafish were anesthetized with 0.02% Tricaine methanesulfonate (Sigma-Aldrich, St. Louis, MO) added to the circulating water of the breeding system. Immediately after confirming the lack of operculum movement, the fish were positioned dorsal side up on a piece of ice covered by a wiper (WYPALL X-60; Kimberly-Clark, Dallas, TX) for the procedure. The tip of the glass micropipette was positioned into the CSF between the hindbrain and the optic tectum. One microliter of a 10-mM 6-OHDA aqueous solution was then injected and the fish were brought back to an aquarium tank filled with water containing 0.1% NaCl. For the control group, vehicle solution was injected. Three days after the 6-OHDA injection, the brains were fixed and subjected to immunofluorescence. Five fish per group were used for the analysis.

### Immunostaining of TH^+^ neurons

Adult fish were sacrificed using circulating water of the breeding system containing 0.1% Tricaine. Brains were removed, explanted, and fixed in 4% paraformaldehyde at 4°C overnight. Subsequently, specimens were embedded in 2% low-melting agarose and 200 μm sagittal or axial sections were collected using a PRO7 microslicer (Dosaka EM, Kyoto, Japan). Floating slices were incubated in 10 mM sodium citrate buffer, pH 8.5, at 80°C for 120 min. After washing in PBS with 1% TritonX-100, the sections were blocked in 2% BSA in PBS/1% TritonX-100 buffer for 30 min. These pretreated sections were incubated overnight at 4°C with a rabbit anti-TH antibody (1:500, AB152; EMD-Millipore, Billerica, MA). After washing with PBS/1% TritonX-100 buffer, the sections were incubated overnight at 4°C with anti-rabbit IgG conjugated to Alexa Fluor 594 (Life Technologies, Carlsbad, CA). The following day, sections were washed in PBS/1% TritonX-100 buffer and analyzed using the A1R+confocal microscope (Nikon, Tokyo, Japan). Ten fish were used for counting TH+ neurons in the posterior tuberculum and the locus coeruleus. Immunostaining of TH^+^ neurons using paraffin sections was performed according to a previously published method [10] and six fish were used for this protocol.

### Statistical analysis

Data was expressed as mean ± standard error of the mean (SEM). Student’s t-test was used to test for statistical significance. Differences with p < 0.05 were considered statistically significant.

## Results

### Optimization of TH^+^ neuron staining

We used microsliced sections of agarose-embedded specimens and found that no signal could be detected when the tissue was immunostained without an antigen retrieval step. We then tested two methods for antigen retrieval using a microwave or hot water bath at 80°C. For both protocols, the sections were immersed in 10 mM sodium citrate buffer, pH 8.5. Both methods enabled successful visualization of TH^+^ neurons, but the microwave method carried the risk of losing sections due to excessive heat and boiling. In contrast, the 80°C incubation was safe, reliable, and produced transparent sections that were optimal for confocal microscopy. Thus, we decided to add the 80°C incubation step in subsequent studies.

**Fig 1:**
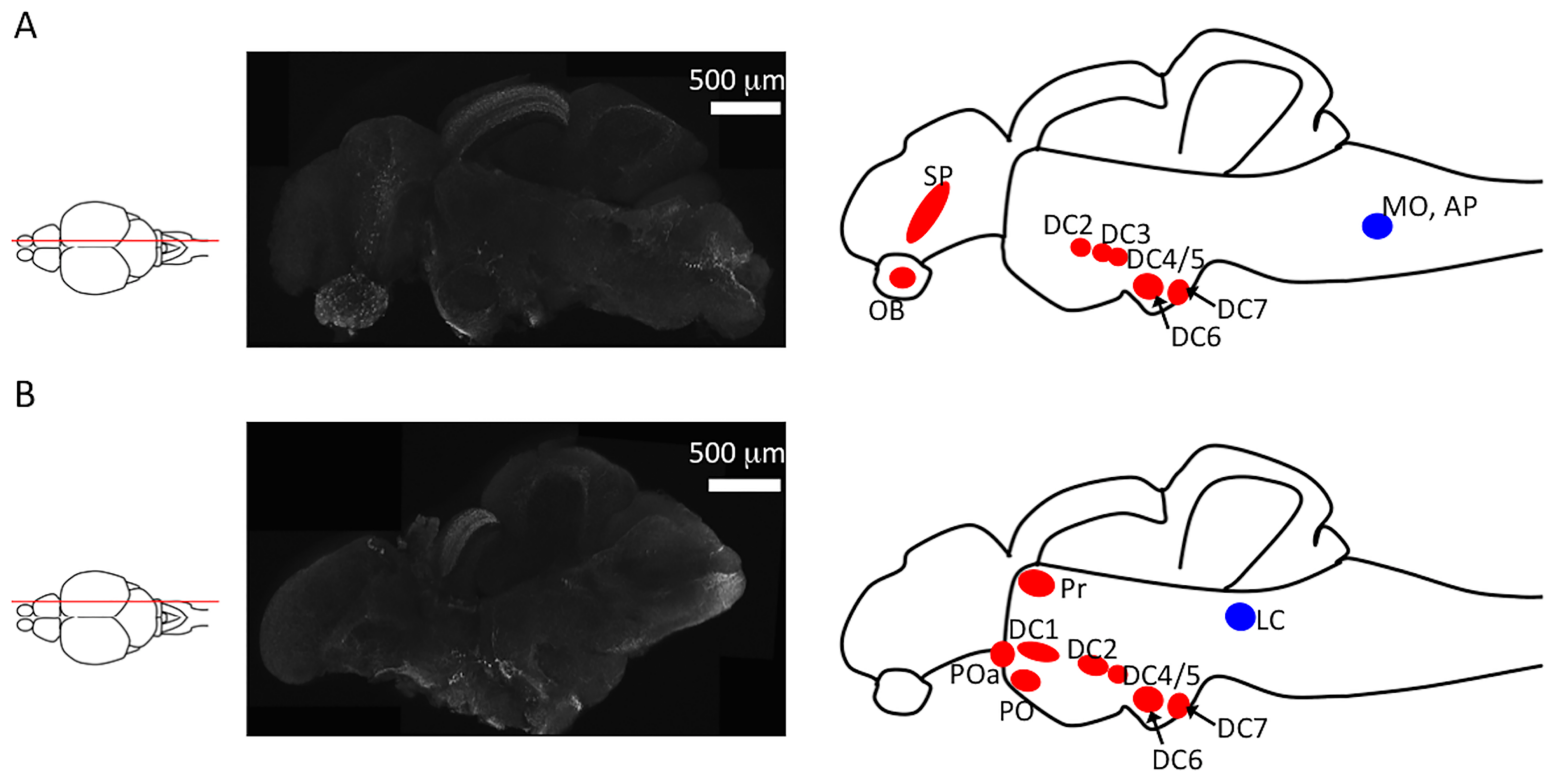
Identification of tyrosine hydroxylase (TH)^+^ neurons in sagittal sections of zebrafish brains. (A) Left panel: an illustration of the sectioning position; middle panel: TH^+^ immunoreactivity in a microsliced section of a 6-month-old zebrafish brain; right panel: TH^+^ neuron clusters found in this section. The red and blue circles indicate dopaminergic and noradrenergic neurons, respectively. (B) Left panel: an illustration indicating the sectioning position. The middle panel is an immunostaining of TH using a microsliced section of 6-month-old zebrafish. The right panel illustrates the TH^+^ neuron clusters in this section. The red and blue circles indicate dopaminergic and noradrenergic neurons, respectively. DC1–7: diencephalic catecholaminergic cluster. Abbreviations: AP, area postrema; LC, locus coeruleus; MO, medulla oblongata interfascicular zone and vagal area; OB, olfactory bulb; PO, preoptic region; POa, anterior preoptic region; Pr, dorsal pretectum; SP, subpallium.

We identified TH^+^ neurons in the olfactory bulb, the telencephalon, the pretectal area, the diencephalon, and the hindbrain of a zebrafish (Fig 1). The distribution of TH^+^ neurons identified by this method was similar to that reported previously in zebrafish and medaka [9,10,37]. Our data indicates that our method can successfully allow the visualization of TH^+^ neurons in the zebrafish brain.

### Counting TH^+^ neurons in the posterior tuberculum and the locus coeruleus

**Fig 2:**
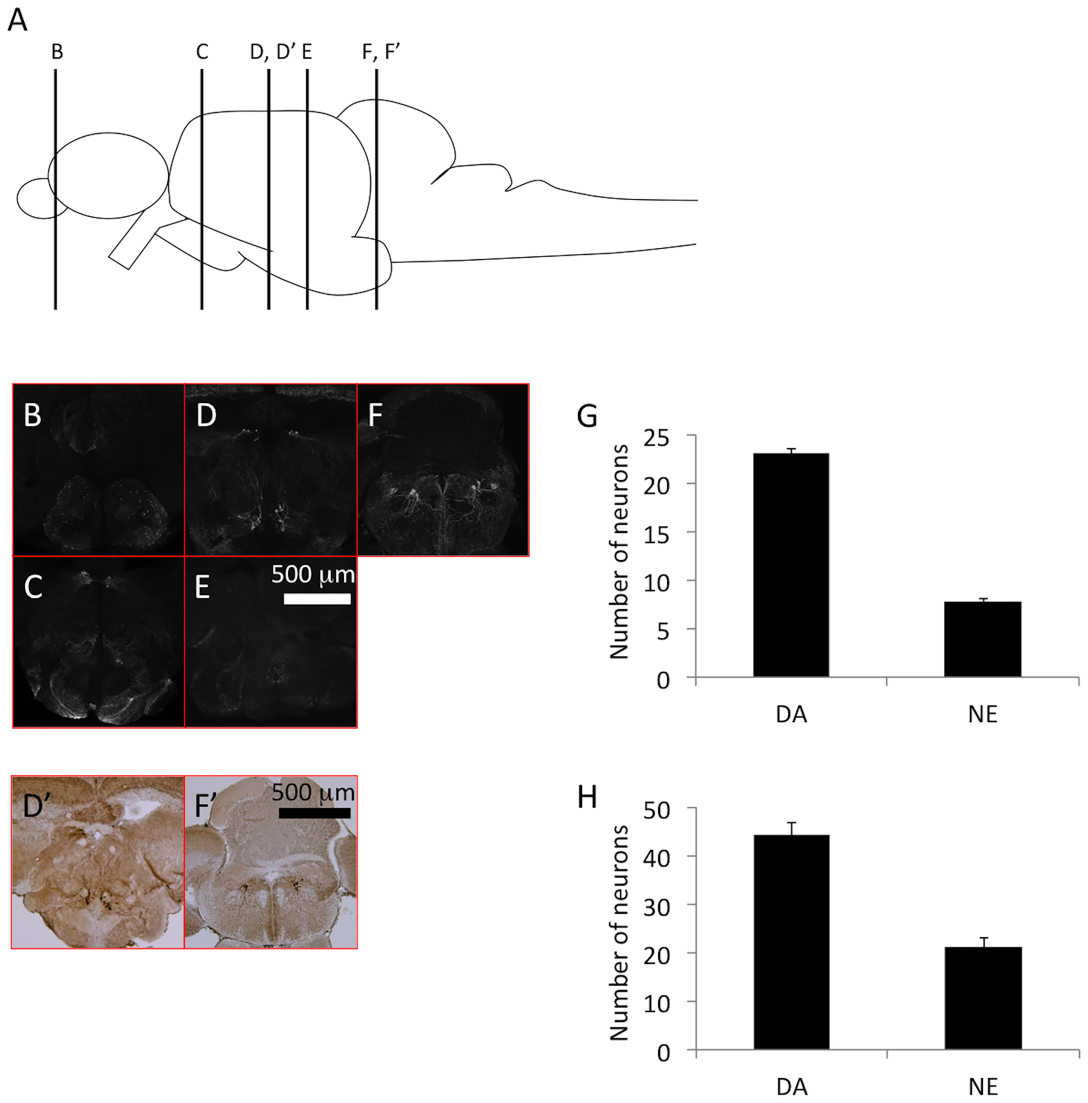
Tyrosine hydroxylase (TH)^+^ neurons in the axial sections of zebrafish brains. (A) The illustration indicates the position of sectioning in panels B–E. (B–E) Immunostaining of TH using a microsliced section of 6-month-old zebrafish. Different rostro–caudal levels show the localization of TH^+^ dopaminergic neurons in the olfactory bulb and subpallium (B); pretectum, posterior periventricular preoptic nucleus, and suprachiasmatic nucleus (C); pretectum, posterior tuberculum, and paraventricular organ (D); and posterior recess of the diencephalic ventricle (E). TH^+^ noradrenergic neurons are distributed in the locus coeruleus (F). (G) The number of TH^+^ neurons in the posterior tuberculum (dopaminergic neurons) and the locus coeruleus (noradrenergic neurons). The bars represent SEM (n = 10). (D’, F’) TH immunoreactivity in a paraffin section of a 6-month-old zebrafish. (H) The counted number of TH^+^ neurons using paraffin sections in the posterior tuberculum (dopaminergic neurons) and the locus coeruleus (noradrenergic neurons). Bars represent the SEM (n = 6).

Patients with PD display a reduction in the dopaminergic neurons of the SNc. We therefore counted the DC2 and DC4 dopaminergic neurons of the posterior tuberculum of zebrafish, which is thought to be equivalent in structure to the human SNc [11,38]. The antigen retrieval step increased the transparency of the sections, allowing us to obtain confocal images of 100–200-μm thick sections. Each section was analyzed from both the rostral and the caudal sides, and the same neuron was identified from both directions to prevent double counting. In this DC2 and DC4 we found 23.1 ± 0.407 neurons with large cell bodies (Fig 2, S1 Movie). We also counted 7.8 ± 0.249 TH^+^ noradrenergic neurons in the locus coeruleus (Fig 2). Counting TH^+^ neurons in the posterior tuberculum and the locus coeruleus yielded highly reproducible data. In contrast, when we counted TH^+^ neurons in 16-μm paraffin sections, we found 44.2 ± 2.651 TH+ dopaminergic neurons and 21.3 ± 1.909 TH+ noradrenergic neurons (Fig 2). The dramatic 1.9–2.7-fold increase in the number of neurons in the paraffin sections suggests that double counting had occurred to a large extent. Furthermore, the standard deviations of the number of TH^+^ neurons were 1.287 and 0.789 for DA and NE, respectively, in 200-μm vibratome sections, and 6.494 and 4.676 for DA and NE, respectively, in 16-μm paraffin sections, indicating increased deviation in the counting when using thinner paraffin sections. In summary, immunostaining of the 200-μm vibratome sections allowed for a more reliable evaluation of the number of dopaminergic and noradrenergic neurons in zebrafish.

### Evaluation of dopaminergic and noradrenergic neurons in zebrafish PD models

**Fig 3:**
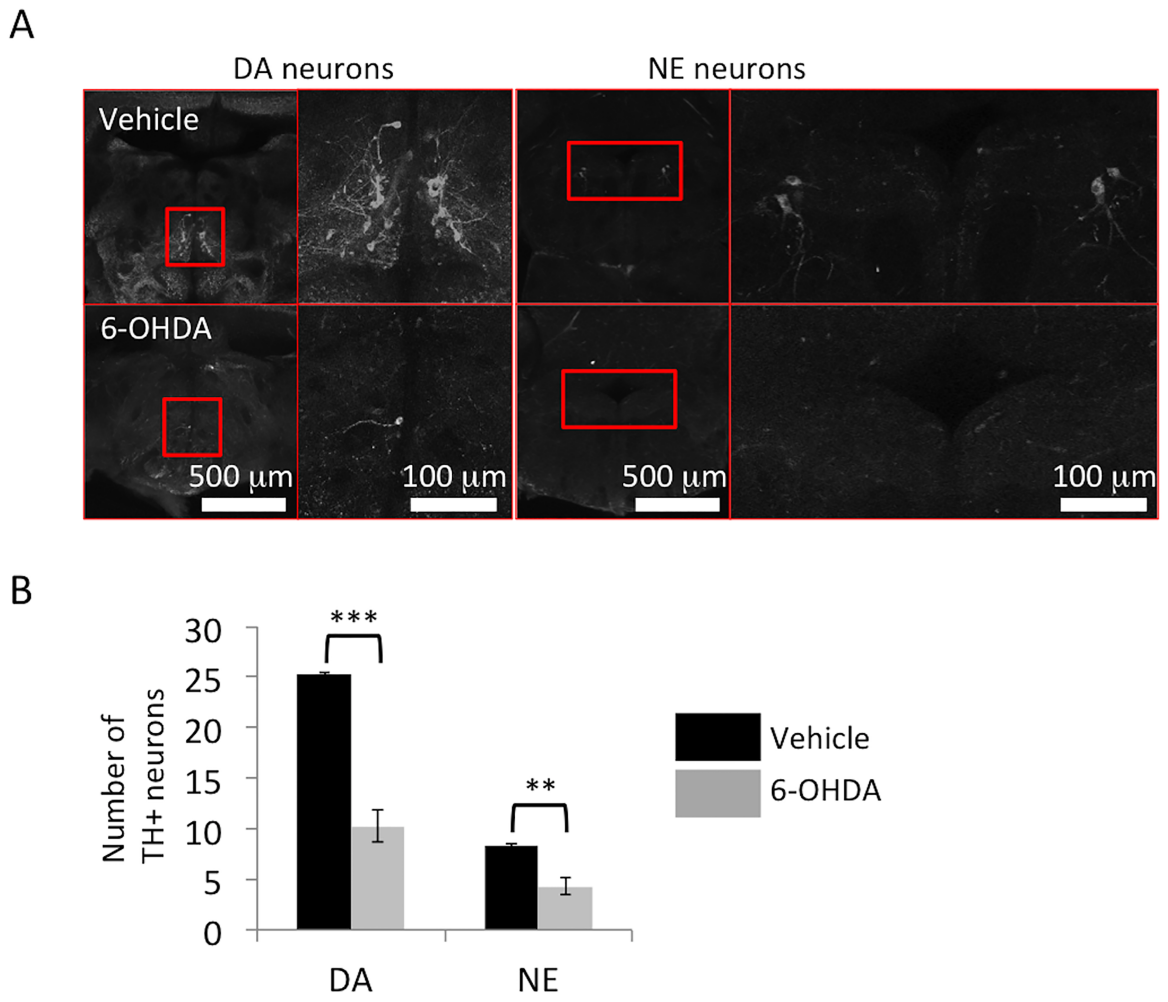
Reduction of tyrosine hydroxylase (TH)^+^ neurons after 6-hydroxydopamine (6-OHDA) administration. (A) Representative images of dopaminergic neurons in the posterior tuberculum and noradrenergic neurons in the locus coeruleus. Right panel: magnified pictures of the red square regions are shown. (B) The numbers of dopaminergic neurons in the posterior tuberculum and noradrenergic neurons in the locus coeruleus are significantly decreased by 6-OHDA treatment in 12-month-old zebrafish; n = 5 fish per group. Bars represent the SEM. **, p < 0.01, ***, p < 0.001.

6-OHDA is a neurotoxin for dopaminergic and noradrenergic neurons. It is taken up by the dopaminergic or noradrenergic neurons via their transporters, and has been used as a tool for producing PD-like lesions in various animal models [39,40]. We therefore administered 6-OHDA to zebrafish and examined whether our counting method was able to accurately evaluate the extent of damage in this model. We used a previously published method [36]—1 μl of 10 mM 6-OHDA or vehicle was injected into the CSF of 12-month-old zebrafish, and the fish were sacrificed 3 days later [36]. Vehicle-treated zebrafish showed no change in the numbers of dopaminergic neurons in the posterior tuberculum and noradrenergic neurons in the locus coeruleus. In contrast, there was a dramatic decline in the number of dopaminergic neurons in the posterior tuberculum and noradrenergic neurons in the locus coeruleus of 6-OHDA-treated zebrafish brains (Fig 3).

**Fig 4:**
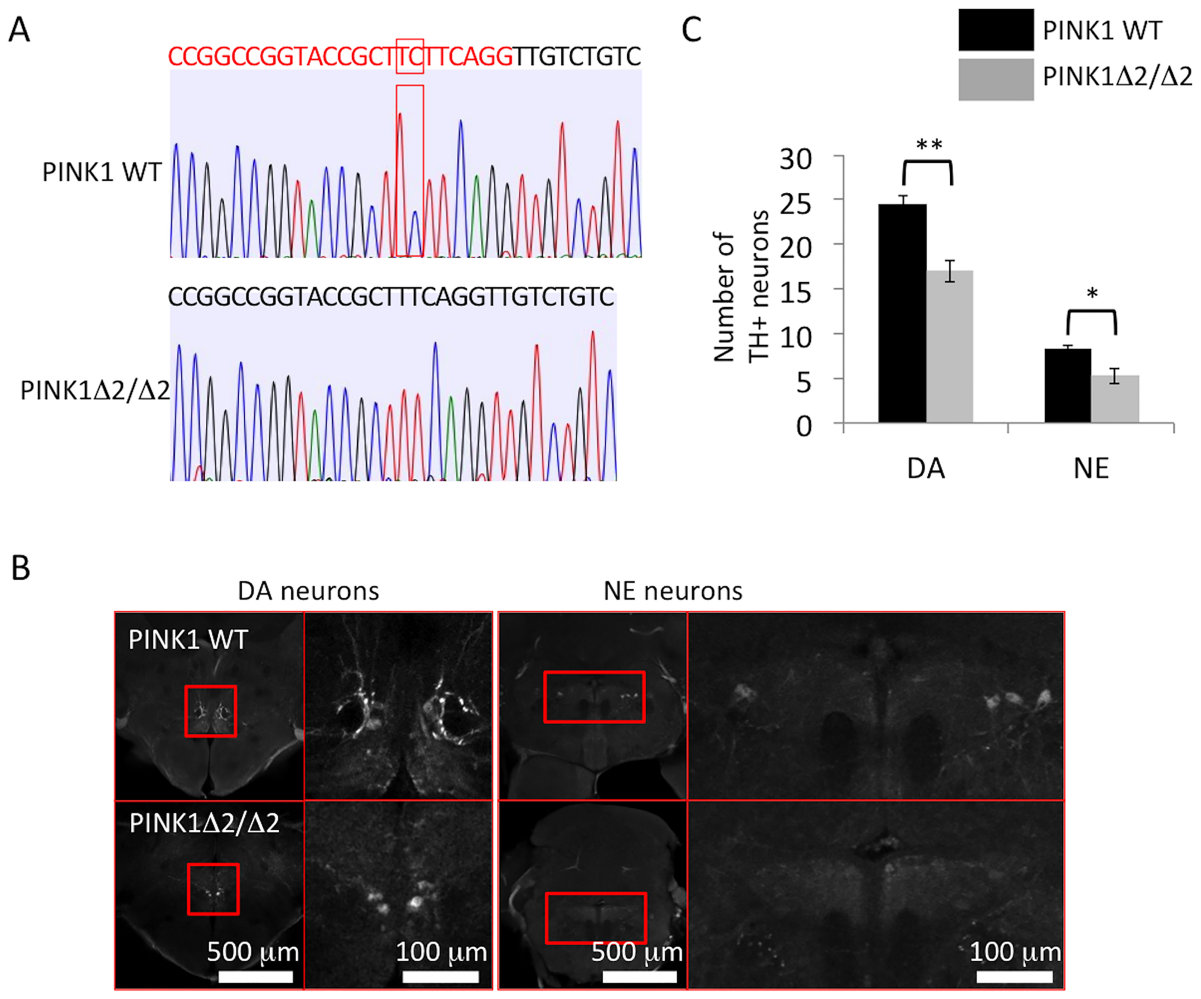
Reduction of tyrosine hydroxylase (TH)^+^ neurons in PINK1-deficient (PINK1-KO) zebrafish. (A) Sequence information of PINK1-deficient zebrafish (c.176_177del, PINK1Δ2/Δ2). The red letter indicates the target sequence of the guide RNA, and the red square indicates the site of the 2-bp deletion. (B) Representative images of dopaminergic neurons in the posterior tuberculum and noradrenergic neurons in the locus coeruleus. Right panel: magnified pictures of the red square regions. (C) The numbers of dopaminergic neurons in the posterior tuberculum and noradrenergic neurons in the locus coeruleus were significantly decreased in PINK1-deficient zebrafish (PINK1Δ2/Δ2) (4-month-old zebrafish); n = 5 fish per group. Bars represent the SEM. *, p < 0.05, **, p < 0.01.

Using the CRISPR-Cas9 system, we next deleted PTEN-induced putative kinase 1 (PINK1); mutations in PINK1 have been found in patients with autosomal recessive forms of familiar PD [34,35]. We raised a mutant line having a 2-bp deletion (c.176_177del, PINK1Δ2/Δ2) in the PINK1 exon. We then compared the number of dopaminergic neurons in the posterior tuberculum and noradrenergic neurons in the locus coeruleus between homozygous mutant (PINK1-KO) and wild type zebrafish at 4 months. Both types of neurons were significantly reduced in the PINK-KO mutants, indicating that PINK1-deficiency caused PD-like neurodegeneration in zebrafish (Fig 4). Taken together, our counting method was applicable for the evaluation of both a toxin-induced PD model and a genetic PD model of zebrafish.

## Discussion

In this study, we proposed a new method for counting TH^+^ neurons in zebrafish and demonstrated reliable counting of these neurons in the posterior tuberculum and the locus coeruleus. Using this method, we showed a significant loss of TH^+^ neurons in these areas in both 6-OHDA-treated and PINK1-deficient (PINK1-KO) zebrafish.

In mouse models, counting dopaminergic neurons in serial sections of the SNc is laborious; therefore, stereological estimation of dopaminergic neurons has often been used [41,42]. Such estimation allows one to count only the sections at specific intervals, reducing the overall cost and labor time. *Drosophila melanogaster* has also been used as a model animal, but discrepancies in the number of TH^+^ neurons have been reported. This may be due to variability introduced by the methods used for TH^+^ staining and counting in the fly brains. Drobysheva *et al*., reported an optimized method for counting fly TH^+^ neurons using thick microsliced sections with agarose-embedded samples [43]. However, in zebrafish and in other fish models, there is currently no standardized method for counting TH^+^ neurons.

Recently, zebrafish and medaka have been used as animal models for PD, creating a need for a reliable method to evaluate the pathophysiology. One of the most prominent pathologies of PD is the degeneration of dopaminergic neurons in the SNc. In zebrafish and medaka, a portion of the dopaminergic neurons in the posterior tuberculum projects to the subpallium [16,24]. These dopaminergic neurons are markedly degenerated in fish that have gene mutations associated with PD [3–6] or that have been exposed to toxins reported to induce PD-like phenotypes in mammals [10,36,44]. Furthermore, ablation of these dopaminergic neurons by lasers reduces spontaneous swimming movements [23]. These findings suggest that the dopaminergic neurons in the posterior tuberculum have similar properties (*i*.*e*., target projection, vulnerability, and physiological functions) as mammalian SNc neurons.

PD is also characterized by movement disorders including resting tremor, muscle rigidity, and reduced movements. However, it is not easy to evaluate resting tremor or muscle rigidity in fish. One could monitor swimming behavior to assess reduction in spontaneous movement, but this assay may not be sensitive enough to detect changes. For example, the ATP13A2 mutant medaka shows degeneration of dopaminergic neurons, but does not exhibit reduced spontaneous swimming [5]. Furthermore, movement disorders of a PD patient may be detected only after the loss of 80% of the neurons in the substantia nigra [45,46]. Thus, it would be important to establish methods to analyze the degeneration of dopaminergic neurons and/or noradrenergic neurons to detect early stages of PD in fish.

Taken together, our method would be useful for the proper evaluation and establishment of zebrafish PD models, and may be applicable to other fish species such as the medaka.

## Supporting information

S1 Movie TH^+^ neurons in the serial sections of zebrafish. The movie shows serial axial sections of the TH^+^ neurons including DC2 and DC4. The movie goes from the rostral section to the caudal.
(AVI)
